# Prevalence and predictors of anxiety and stress among Jordanian women following hysterectomy: an observational multicentre study

**DOI:** 10.1186/s40359-025-02623-1

**Published:** 2025-03-26

**Authors:** Rasmieh Al-amer, Maha Atout, Malakeh. Z. Malak, Ahmad Ayed, Wafa’Mousa Othman, Mohammad Y.N. Saleh, Lobna Harazne, Amira Ali, Sue Randall

**Affiliations:** 1 Faculty of Nursing, Yarmouk University, Irbid - Jordan, Irbid, P.O.BOX (3678), 11953 Jordan; 2 Pediatric Nursing, Philadelphia University| Faculty of Nursing, Jarash Road, P.O. Box 19392, Amman, Jordan; 3 Faculty of Nursing, Al-Zaytoonah University of Jordan, Amman, Jordan; 4 Paediatric health nursing, Arab American University, Jenin, Palestine; 5 Clinical Nursing Department, Faculty of Nursing, Hashemite University, Amman, Jordan; 6 Clinical Nursing Department, The University of Jordan, School of Nursing, Amman, Jordan; 7 Psychiatric and mental health nursing, Arab American university, Jenin, RN Palestine; 8 Department of Psychiatric Nursing and Mental Health, Faculty of Nursing, Alexandria University, Alexandria, Egypt; 9 The University of Sydney, Faculty of Medicine and Health, Susan Wakil School of Nursing and Midwifery, Sydney, Australia

**Keywords:** Hysterectomy, Anxiety, Stress, Body appreciation, Social support

## Abstract

**Background:**

Jordan is a collectivist society where fertility is rated highly. Hysterectomy, therefore, has the potential to negatively impact a woman’s standing in a collectivist society leading to increased levels of anxiety and stress.

**Purpose:**

To assess the impact of hysterectomy on the levels of anxiety, stress, body appreciation, and social support among women.

**Methods:**

A cross-sectional design was utilized to recruit 251 women post-hysterectomy. The study used the Depression, Anxiety, and Stress Scale (DASS), the Enriched Social Support Instrument (ESSI), and the Body Appreciation Scale (BAS).

**Results:**

Sexual difficulties were experienced by the majority. Severe anxiety was reported by 39% with around 89% of women reporting stress that was moderate or higher. Overall, participants had moderate levels of body appreciation and a high level of perceived support. Sexual problems, body appreciation, stress, post-surgery duration, and social support predicted the levels of anxiety, with both a desire for more children and longer post-surgery durations heightening both anxiety and stress.

**Conclusion:**

Hysterectomy negatively impacts women’s mental health, leading to high levels of anxiety and stress. Body appreciation and social support are important facets in buffering the consequences of hysterectomy. A culturally sensitive healthcare addressing the individual needs of women in collectivist communities is paramount.

**Supplementary Information:**

The online version contains supplementary material available at 10.1186/s40359-025-02623-1.

## Introduction

Hysterectomy is a common gynecological procedure involving the removal of the uterus [1]. While often necessary for medical reasons, hysterectomy can lead to significant psychological challenges, including anxiety and stress; these factors are frequently overlooked. The incidence of hysterectomy varies from 2.13–3.62/1,000 in Germany to 5.4/1,000 in the United States [2]. In the developing world, it ranges from 1.31/1,000 deliveries in Egypt [3] to 113.5/1,000 deliveries in India [4]. In Jordan, the incidence of hysterectomy for benign conditions varies significantly, with rates reported between 0.24 and 8.7 per 1,000 deliveries in a tertiary hospital located in a major governorate (an area controlled by a governor), which serves patients from across the country [5]. Similarly, a recent study conducted in a northern governorate of Jordan reported that the incidence of peripartum hysterectomy is 1.38 per 1,000 births, reflecting that regional variations were likely influenced by differences in clinical practices and institutional resources [6].

Approximately 90% of hysterectomies are for benign conditions such as fibroid uterus and dysfunctional uterine bleeding, with malignancy being another indication [1, 7–9]. Hysterectomy is linked to a mortality rate of 1 in 1000 [2] and can result in physical and psychological complications. Physical complications include bleeding, infection, and sexual difficulties [10, 11]. Psychological difficulties involve depression, anxiety, and stress [11, 12].

Hysterectomy has long been linked to psychological reactions, with early reports indicating a high probability of poor mental health, including psychosis within three years [13]. Women often view the uterus as a core element of their femininity symbolizing youth, vitality, and childbearing [11, 14], leading to various psychological issues post-hysterectomy [11, 15, 16]. Women may experience reduced self-confidence, poor body image, relationship issues, and a decline in quality of life [10, 14, 17]. For those wanting more children, hysterectomy can precipitate significant psychological changes such as severe anxiety and stress [11].

Studies in Low- and Middle-Income Countries (LMICs) with collectivist cultures, such as Egypt, Pakistan, and Turkey, have reported high levels of anxiety and depression before and after hysterectomy, often linked to feelings of lost femininity and childbearing capacity [14, 18, 19]. However, other studies from Egypt and India have shown positive outcomes, with women reporting improved quality of life except in sexual function [20, 21]. Some women reported that hysterectomy alleviates their chronic pain, and other gynecological problems, leading to improvement in the quality of their life [21–23]. These conflicting results call for more studies, specifically in collectivist communities.

In the Arab world, particularly in Jordan, data on the psychological outcomes of hysterectomy are scarce. Sociocultural factors, such as mental health stigma and limited resources, particularly in these communities, may exacerbate the impacts of anxiety and stress in women post-hysterectomy. Additionally, limited access to specialized psychological care and societal taboos surrounding the discussion of mental health issues result in a more complicated mental health status [24]. Understanding the prevalence and predictors of anxiety and stress among women undergoing hysterectomy is critical to addressing these mental health issues in collectivist communities.

### Jordanian context

Jordanian women, who are of Arabic descent, live in a collectivist society where fertility is highly valued and tied to identity and social status [24]. In this context, women unable to bear children may face social marginalization and devaluation, as noted in studies exploring fertility and cultural perceptions in the region [25, 26].

In this culture, children are viewed as a vital investment in the future, as they are expected to care for their parents as they age [27, 28]. Hence, in a collectivist society, a hysterectomy, which renders complete infertility, can lead to extreme psychosocial repercussions, as a woman’s worth is deeply tied to her ability to bear children.

In the Jordanian community, where polygamy is allowed, a woman unable to bear children may fear that her husband might seek another wife, exacerbating their feelings of loss and vulnerability [29–31]. Overall, this cultural framework highlights the multifaceted challenges encountered by women post-hysterectomy, in which physical and societal factors shape their mental well-being [32].

### Theoretical framework

The Biopsychosocial Model (BPS) offers a framework to understand how biological, psychological, and social factors collectively impact health [33]. This model suggests that wellness and illness stem from the intersection between these factors. Additionally, it integrates cognitive appraisal, highlighting how individuals’ perceptions of biological threats influence their social and emotional responses. Holistic healthcare plans that address physical, psychological, and social needs are vital [33, 34]. In hysterectomy, the removal of the uterus and its related complications, such as hormonal issues and sexual difficulties, can result in anxiety and stress [35]. These biological changes are often compounded by psychosocial factors such as how women cognitively appraise their bodies and cope with hysterectomies [35, 36]. Negative appraisal can exacerbate their mental health, while positive appraisal can alleviate it.

Social factors, such as social support and cultural norms, also play a crucial role in shaping mental health outcomes post-hysterectomy [33]. In collectivist societies where fertility is valued, women undergoing hysterectomy may feel inadequate, leading to low body appreciation, and increased anxiety and stress [31, 32]. High social support can provide emotional comfort and promote adaptive health behaviors [37, 38].

Overall, the BPS model emphasizes the value of a holistic approach, considering the intersection of biological, social, and psychological factors for individualized care plans [33].

## Methods

This study aimed to investigate the influence of hysterectomy on the levels of anxiety, stress, body appreciation, and social support among women who underwent hysterectomies, hence, the study addresses the following research questions:

1. What are the levels of stress, anxiety symptoms, body appreciation, and social support among Jordanian women who underwent hysterectomy?
2. What are the associations between the levels of anxiety, stress, body appreciation, and social support and sociodemographic data among Jordanian women post-hysterectomy?
3. What are the predictors of stress and anxiety among Jordanian women who underwent hysterectomy?

### Population

The population comprised all women who underwent hysterectomy for benign conditions. The inclusion criteria were (a) women who had undergone hysterectomy for non-cancerous reasons; (b) no cognitive impairment or diagnosed mental illness, based on medical reports or self-reporting; (c) able to read and write in Arabic; (d) aged 18 to 50 years. This age range was selected as it encompasses many benign condition-related hysterectomies observed in Jordan [39]; and (e) no significant mental health event in the past six months; (f) the surgery took place between two months and two years before data collection (duration post-surgery). The post-surgery duration of two years was set as an inclusion criterion to minimize the confounding effects of serious long-term complications of hysterectomy, which often occur within three years of the procedure. These complications include cardiovascular events, certain cancers, early ovarian failure, menopause, and pathological depression [40]; j) provided informed consent. Exclusion criteria included (a) hysterectomy for cancer; (b) women on hormone replacement therapy; (c) those who had attained menopause; and (d) any significant traumatic event in the past six months.

### Design, setting, and sampling

In this cross-sectional study, data from hospitals affiliated with the Ministry of Health in Jordan (MOH), specifically in Amman (Jordan’s capital) were used. Amman is the largest city by area and population, with around 4,500,700 residents according to the Jordanian Statistics Department [41]. Thus, women recruited from this governorate are representative of Jordanian women. The sample size calculation was conducted using G*Power 3.0.10. The calculation parameters were set at 0.95 power and 0.05 significance levels, with 12 selected predictors using regression. The sample size calculation determined the need for 184 participants. Three hundred questionnaires were distributed, yielding 275 responses, of which 251 were fully completed and subsequently included in the analysis.

### Study measures

We used a structured self-reported survey to collect data from participants. This survey included four components: a demographic questionnaire and three validated scales, the anxiety and stress subscales of the DASS [42–44]; the Enriched Social Support Instrument (ESSI) [45, 46], and the Body Appreciation Scale (BAS) [47]. The demographic questionnaire was developed based on existing literature [12, 39, 48].

### Depression, anxiety and stress scale (DASS)

The current study used Depression, Anxiety, and Stress Scale 21-item (DASS-21), a 21-item comprising three subscales developed to assess emotional states: depression, anxiety, and stress [42]. Each subscale includes 7 items anchored in a four-point Likert scale from 0 (did not apply to me at all) to 3 (applied to me very much, or most of the time). The scores of each subscale were added together for the corresponding items. For more details about the subscales scoring system see appendix 1. This study utilized the Arabic version of the DASS, which has been extensively used among Jordanian populations. The scale demonstrates strong reliability, with a Cronbach’s alpha of 0.95 for the total DASS [43, 44]. Specifically, among Jordanians, the scale has shown Cronbach’s alpha values of 0.94 for the depression subscale, 0.90 for the anxiety subscale [49], and 0.89 for the stress subscale [50].

### Enriched social support instrument (ESSI)

The levels of social support were assessed using the Arabic translation of the Enriched Social Support Instrument (ESSI); [51]. The ESSI scale is composed of seven statements. The first six statements use a five-point Likert scale ranging from 8 to 34 with higher scores indicating higher levels of social support. This scale is scored as follows, a)1 = none of the time; b) 2 = a little of the time; c) 3 = some of the time; d) 4 = most of the time; e) 5 = all the time. The seventh item is a yes/no question in which yes is scored as 4 and no is scored as 2 [45]. The Arabic version of the Enriched Social Support Instrument (ESSI) was validated in a previous thesis through Exploratory Factor Analysis (EFA), which confirmed its unidimensional structure with robust psychometric properties (KMO = 0.82; Bartlett’s Test: χ² = 597.577, *p* < 0.001). All seven items were loaded onto a single component, explaining 59.25% of the variance, consistent with the original scale [45]. Furthermore, the scale has been successfully utilized in studies involving Jordanian populations without changing its items or sub-dimensions [46].

### Body appreciation scale (BAS)

The BAS consists of six different body appreciation subscales. Example items include “How good I feel about my body depends a lot on whether people consider me good-looking” and “How good I feel about my body depends a lot on what my body can do physically [47]. Participants were asked to complete an Arabic-translated version of the BAS-2. This 10-item scale assesses acceptance of, respect, and care for one’s body and protection from unrealistic beauty standards. Items are rated on a 5-point scale, ranging from 1 (never) to 5 (always), and an overall score is computed as the mean of the 10 items with higher scores on this scale being reflective of the greater body appreciation. The BAS-2 has shown satisfactory reliability and validity in samples from diverse socio-cultural contexts [47]. Psychometric testing of the Arabic version of the Body Appreciation Scale-2 (BAS-2) was conducted among Arab participants. Exploratory Factor Analysis (EFA) and Confirmatory Factor Analysis (CFA) confirmed its unidimensional factor structure, consistent with the original English validation of the 10-item BAS-2. All 10 items were loaded onto a single factor for male and female respondents [52].

### Pilot testing

The questionnaires were pilot tested among 30 women who met the study’s inclusion criteria and attended the same health setting. This pilot aimed to examine feasibility, including the time required to complete the survey. A blank sheet was provided for the participants to offer feedback, which was minimal and incorporated into the final version. Data from the pilot were excluded from the final report to prevent data contamination.

### Data collection procedures

After obtaining ethical approval, we recruited patients and collected data from hospitals affiliated with the Ministry of Health in Amman. The nursing manager received a detailed explanation of the purpose of the study, and permission was requested to contact potential participants.

Subsequently, a poster was hung on the wall of the gynecological clinic at each participating hospital to advertise the study; the poster included the first researcher’s detailed contact information. Patients who contacted the researchers were screened for eligibility, and those who were eligible received a detailed explanation of the study’s purpose and were informed about their right to withdraw from the study at any point without penalties. Then, in the hospital setting, each participant received the study questionnaires and was asked to enclose them in the envelope provided and return them directly to the primary researcher or to leave them in the reception area, where the researcher later collected them. The women completed the questionnaires in a private and quiet room at the clinic while waiting for their appointments with their physicians. This arrangement was made to ensure comfort and confidentiality during data collection.

### Statistical analysis

For data analysis, the Statistical Package for Social Sciences (SPSS) version 24 was utilized. Descriptive statistics summarized participants’ demographic, clinical, and socioeconomic characteristics. Continuous variables were reported as means and standard deviations (SD), while categorical variables were presented as frequencies and percentages (n, %). Pearson’s correlation coefficient (r) was used to assess the strength and direction of relationships between continuous variables, and Point-Biserial Correlation (p.b.r.) was employed for dichotomous categorical variables, with significance levels set at *p* < 0.05.

A regression model was performed to identify predictors of anxiety and stress among study participants. Results were reported using unstandardized coefficients (B), standardized coefficients (β), 95% confidence intervals (CI), and P values (*p* < 0.05). Model fit was evaluated through R-squared (R²) and adjusted R-squared values.

## Results

**Table 1:** Demographic and clinical characteristics of study participants

Variables	*n* (%)
Age, Mean (SD): 43.69 (7.14); Range: 21–50 years	
Marital Status	
Married	188 (74.90)
Widow	34 (13.54)
Divorce	12 (4.78)
Single	17 (6.77)
Levels of Education	
Primary	42 (16.73)
Secondary	113 (45.01)
Tertiary	96 (38.24)
Number of Alive Children	
No Children	35 (13.94)
One Child	12 (4.78)
Two Children	41 (16.33)
Three Children	47 (18.72)
≥Four Children	116 (46.21)
Desire for More Children	
Yes	61 (24.30)
No	190 (75.96)
Work Status	
Yes	86 (34.26)
No	150 (59.76)
Post-Surgery Duration	
2-Month– 6 Month	158 (62.94)
> 6 Month– 2 Years	93 (37.05)
Indication for Surgery	
Uterine Fibroid	137 (54.58)
Menorrhagia	86 (34.26)
Dysfunctional Uterine Bleeding	20 (7.96)
Sexual Problem	
Yes	147 (58.56)
No	38 (15.13)
Body Mass Index (kg/m²)	
> 18.5	5 (1.99)
18.5–24.9	65 (25.89)
25–29.9	84 (33.46)
≥ 30	97 (36.64)
Family Income in Jordanian Dinar, JD	
< 500	168 (66.93)
500–1000	59 (23.50)
> 1000	8 (3.18)

Number of participants (251)

SD: Standard deviation; Body Mass Index was classified based on the WHO definition

Family income: Each Jordanian Dinar (JD) equals 1.41 US Dollars

n (sample size) in this table was calculated based on the available data

The study sample consisted of 251 women, with an average age of 43.69 years (SD = 7.14), ranging from 21 to 50 years. Approximately 75% were married, 45.0% had completed secondary education, 46.2% had four or more children, and 34.3% were employed. The primary indications for surgery were uterine fibroid (54.6%) and menorrhagia (34.3%). More details are depicted in Table 1.

Sexual difficulties reported by the study participants post-hysterectomy.

**Fig. 1:**
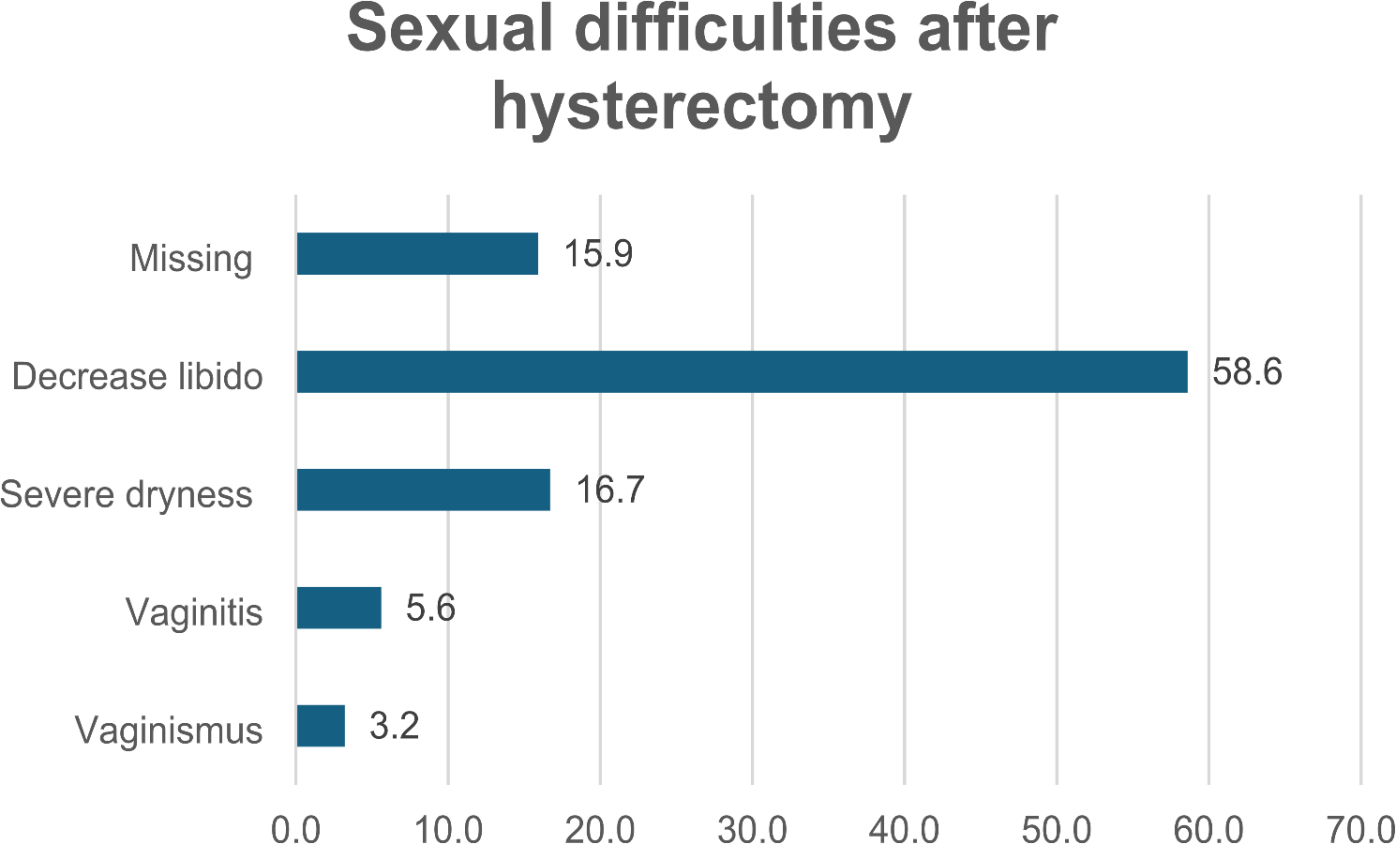
Percentage of study participants experiencing sexual difficulties after hysterectomy

Figure 1 shows the sexual difficulties reported by the study participants post-hysterectomy. Approximately 60% reported a decrease in libido, 16.7% experienced severe dryness, 5.6% experienced vaginitis, and 3.2% reported vaginismus. These values were calculated based on the available data.

**Table 2:** Anxiety and stress levels among Jordanian women post-hysterectomy

Anxiety		Stress	
Category: Range	n (%)	Category: Range:	n (%)
No Anxiety: 0–7	80 (31.87)	No Stress: 0–14	36 (14.34)
Mild Anxiety: 8–9	18 (7.17)	Mild Stress: 15–18	101 (40.23)
Moderate Anxiety: 10–14	56 (22.31)	Moderate Stress: 19–25	94 (37.45)
Severe Anxiety: 15–19	34 (13.54)	Severe Stress: 26–33	19 (7.56)
Extremely Severe Anxiety: ≥20	63 (25.09)	Extremely Severe: ≥34	1 (0.39)

Number of participants (251)

Table 2 illustrates that 22.3% reported having moderate anxiety, 13.5 experienced severe levels of anxiety, and 25.1% reported extremely severe anxiety (scores ≥ 20). Regarding stress, 40.2% reported mild levels, while 37.5% and 7.6% experienced moderate to severe stress respectively.

**Table 3:** Characteristics of standardized measures

Standardized Scales; Range:	Mean (SD)
Anxiety Subscale: 0–42	13.1 (9.5)
Stress Subscale: 5–42	19.5 (10.3)
Body Appreciation Scale: 14–46	26.2 (6.5)
ENRICH Social Support Instrument (ESSI): 8–34	21.6 (7.3)

SD: Standard Deviation

Table 3 shows that many participants had moderate levels of anxiety given the spread of scores across the full range of the scale. The average stress score was 19.5 (SD = 10.3), with a range of 5–42 indicating moderate stress levels. The BAS has a mean score of 26.2 (SD = 6.5), ranging from 14 to 46, suggesting mild overall body appreciation. However, the range of body appreciation scores suggests significant variability, indicating that some participants were highly likely to experience low levels of body appreciation. The mean score of the ENRICHED Social Support Instrument (ESSI) was 21.6 (SD = 7.3), ranging from 8 to 34, indicating a generally high level of perceived social support among participants.

**Table 4:** Correlation between anxiety, stress, and study-related variables among Jordanian women post- hysterectomy

Variable	Anxiety		Stress	
	*r*	*p*	*r*	*p*
Age	**−0.208**	0.001	**-0.259**	0.001
Stress Levels	**0.634**	0.001	1.000	1.000
Post-Surgery Duration	**0.847**	0.001	**0**.**485**	0.001
Body Appreciation Levels	**-0.581**	0.010	**-0.246**	0.010
Social Support Levels	**0.336**	0.010	**-0.216**	0.010
Anxiety Levels	1.000	1.000	**0.634**	0.001
	*p.b.r*	*p*	*p.b.r*	*p*
Work Status	0.095	0.123	-0.003	0.959
Marital Status	-**0.146**	0.010	-0.077	0.225
Desire for More Children	**0.486**	0.001	**-0.385**	0.001
Number of Children	**-0.235**	0.001	**-0.218**	0.001
Family Income	-0.039	0.535	-0.057	0.365
Sexual Difficulties	-0.052	0.520	**-0.146**	0.021
Levels of Education	0.083	0.190	0.053	0.407

p: p-value

r: Pearson’s correlation coefficient

p.b.r: Point-biserial correlation coefficient (used where applicable)

* Correlation is significant at the 0.05 level (2-tailed)

** Correlation is significant at the 0.01 level (2-tailed)

Table 4 illustrates that there was a negative correlation between age and both anxiety (*r* = -0.208, *p* = 0.001) and stress (*r*=-0.259; *p* = 0.001) indicating that older participants tend to have lower levels of anxiety and stress. There is a strong positive correlation between stress and anxiety levels (*r* = 0.634, *p* = 0.001), highlighting that individuals with higher anxiety also tend to experience higher stress. Body appreciation was negatively correlated with both anxiety (*r* = -0.581, *p* = 0.01), suggesting that higher body appreciation was associated with lower anxiety and stress and that social support was negatively associated with anxiety (*r* = -0.336, *p* = 0.01). The number of children was negatively correlated with anxiety (*r* = -0.235, *p* = 0.001) and stress (*r* = 0.218^**^, *p* = 0.001). More details are presented in Table 4.

**Table 5:** Predictors of anxiety among Jordanian women Post-Hysterectomy

Variables	B		β	t	*p*	95.0% CI for B
Desire for More Children	−4.066	1.081	−0.185	−3.762	< 0.001	[− 6.195, − 1.937]
Sexual Problems	−1.071	0.511	−0.077	−2.095	0.037	[− 2.077, − 0.064]
Body Appreciation	1.231	0.095	0.656	12.993	< 0.001	[1.044, 1.417]
Stress	0.394	0.090	0.215	4.388	< 0.001	[0.217, 0.571]
Social Support	−1.966	0.635	−0.114	−3.095	0.002	[− 3.217, − 0.715]
Post-Surgery Duration	−15.936	0.695	−0.813	−22.915	< 0.001	[− 17.306, − 14.566]

Multiple Linear Regression; Model Summary: R² = 0.87, Adjusted R² = 0.75, Standard Error of the Estimate = 4.82, F = 79.76, df for F-statistics (F (9,251)

B: Unstandardized Coefficients; β: Standardized Coefficients; CI: Confidence Interval; t: t-value; p: p-value

Dependent Variable: Anxiety. Number of participants: 251.

As illustrated in Table 5, the multiple regression model was statistically significant (F change = 79.762, df = 9, *p* < 0 001). This model explains around 75% of the variance in total anxiety score (adjusted R Square = 0.750). Furthermore, the table shows that the desire for more children and sexual problems were significant predictors of the levels of anxiety (β = -4.066, *p* < 0 0.001) and (β = -1.071, *p* = 0 0.037) respectively. Additionally, body appreciation levels, the levels of stress, post-surgery duration, and social support predicted the levels of anxiety (β = 1.231, *p* < 0 0.001), (β = 0.394, *p* < 0.001), (β = -1.966, *p* < 0 0.002), and (β = 15.936, *p* < 0.001), respectively.

**Table 6:** Predictors of stress among Jordanian women who underwent hysterectomy

Variables	B		β	t	*p*	95.0% CI for B
Anxiety Total	0.189	0.043	0.347	4.394	0.000	[0.104, − 0.274]
A desire for More Children	1.581	0.758	0.132	2.087	0.038	[0.089, − 3.073]
Post-Surgery Duration	−2.113	0.911	−0.198	−2.320	0.021	[− 3.907, − 0.319]

* Multiple Linear Regression; Model Summary: R Square: 0.530, Adjusted R Square: 0.508, Standard Error of the Estimate: 3.61, F Change: 24.42, df for F-statistics (F (9,251)

B: Unstandardized Coefficients; β: Standardized Coefficients; CI: Confidence Interval; t: t-value; p: p-value

Dependent Variable: Stress

Table 6 shows that the regression model for stress was statistically significant (F change = 24.42, df = 9, *p* < 0 0.001), explaining 50.8% of the variance in stress (adjusted R Square = 0. 508). Anxiety levels (β = 0 0.189, *p* < 0 0.001) and post-surgery duration (β = -2.113, *p* < 0.021) significantly predicted stress. Additionally, the desire for more children is associated with higher levels of stress (β = 1.581, *p* = 0.038).

Discussion and conclusions.

This study investigated the influence of hysterectomy on anxiety, stress, body appreciation, and social support among women post-hysterectomy; and identified the determinants of anxiety and stress. The sample consisted of Jordanian women who had undergone hysterectomy for benign conditions. The findings showed that women experienced high levels of anxiety and stress, and mild body appreciation. Women in this study reported good social support indicating adequate post-hysterectomy support. The intersection between these factors is crucial for understanding how a biological or somatic event could influence the psychosocial aspects of women following hysterectomy.

A key finding of this study is that most of the study participants experienced sexual difficulties, with decreased libido being the most frequently reported one. A possible explanation could be related to both physical and psychological aspects, including possible nerve damage and reduced pelvic blood flow, impairing sexual response [12]. Hysterectomy can also alter the perception of femininity and sexual identity and image issues affecting sexual desire [12]. However, evidence regarding sexual dysfunction post-hysterectomy is conflicting. For example, some studies reported that decreased libido and sexual difficulties were common among women who have undergone hysterectomy [10, 12], while another study reported positive health outcomes aside from sexual function [20]. Conversely, a study found no significant association between hysterectomy and a reduction in sexual function in benign conditions [53]. The inconsistencies in the literature regarding sexual dysfunction post-hysterectomy may be attributed to heterogeneity among studies. Variations in methodology, such as differences in sample size, participant characteristics, and the timing of assessments post-hysterectomy, are likely to contribute to these conflicting findings. This inconclusive evidence contributes to a lack of counselling on sexual function following a hysterectomy [53].

The psychological impact of hysterectomy extends beyond physical changes, particularly concerning body image. Women’s perceptions of their body appearance following hysterectomy can negatively affect their sexual identity and confidence. Additionally, cultural factors play a significant role; in some collectivist societies, a woman’s sense of identity and self-worth is closely tied to her role as a mother, which may further exacerbate the psychological consequences of hysterectomy [37].

It is worth mentioning that the psychological effects of hysterectomy on sexual identity are further influenced by gendered expectations surrounding femininity and sexual health. To illustrate, a woman’s sexual identity is linked to her reproductive capacity; hence, losing this may lead to feelings of diminished sexual desirability, loss of femininity, and low self-worth, particularly in collectivist cultures where motherhood is highly valued [12, 14, 15].

In addressing the psychological status among women who have undergone hysterectomy, this study indicated that more than one-third of the study subjects experienced severe to extremely severe anxiety, while around 40% had moderate stress. Our results suggest mental health complexities among this cohort. Our findings should be viewed considering the distinct nature of anxiety and stress. Although anxiety and stress overlap, they are distinct psychological constructs. Anxiety involves ongoing concerns about future events and uncertainties, while stress arises from immediate demands that exceed the person’s resources at the same moment [54, 55]. Hence, it is plausible that the loss of the reproductive organs complicates the psychological status of women. We hypothesized that this could be exacerbated by culture. For example, in collectivist Arabic culture, infertile women could be subjected to diminished status and social exclusion, leading to high levels of anxiety and stress [25].

In line with our study, hysterectomy as a biological threat has been reported to complicate the psychosocial aspects of women’s lives [10]. Depression, anxiety, and stress were common post-hysterectomy [11, 12]. Developing countries with collectivist cultures, such as Egypt, Pakistan, and Turkey also show that women experience elevated anxiety and depression before and after hysterectomy [12, 14, 18, 19].

Several factors influenced anxiety levels among the study subjects, younger women and those with higher stress exhibited more anxiety. The duration of post-surgery played a role, with shorter time after surgery associated with higher stress, this could be due to emotional adjustment to uterine loss. For example, the association between a shorter post-surgery duration and increased stress may reflect a temporary adjustment phase during the early recovery period, rather than a lasting psychological effect. Over time, it is plausible that stress levels decrease as individuals adapt to their new circumstances [56]. Our findings support previous studies showing anxiety and stress is common post-hysterectomy, particularly among younger women [10, 14, 19]. Despite this significant correlation between age and anxiety and stress, age was not a predictor of these psychological reactions in our study.

Those who viewed their bodies more positively after surgery were highly likely to have a lower level of anxiety [35, 36]. Our findings are consistent with other research indicating negative body image post-hysterectomy can lead to reduced self-confidence, poor body image, relationship issues, sexual difficulties, and a decline in quality of life [15, 17].

Social support, viewed as the social factor in the BPS, emerged as a significant predictor of anxiety, with higher levels of support resulting in lower levels of anxiety; suggesting that social support could balance the emotional impact of losing the uterus. Marital status and the number of children also were equally significant factors in mitigating the negative psychological status post-hysterectomy. A supportive husband and children can provide timely support when needed. In addition to that, women with more children might feel more secure despite the inability to have more. On the other hand, the inability to bear children added another layer of emotional stress, exacerbating anxiety and potentially worsening their psychological status. Our findings mirror the importance of social factors in mental health outcomes following a hysterectomy [21, 57], consistent with the BPS model, which focuses on the intersection between biological, psychological, and social factors.

Being fertile and post-surgery duration emerged as significant influencing factors for anxiety and stress. To illustrate, anxiety levels predict stress levels, indicating a direct correlation between these two variables; although these variables are distinct, they are related and interconnected. Hence, managing anxiety is a vital component of managing stress and vice versa. Women who desired more children experienced higher levels of anxiety and stress. Thus, these results could be explained by a collectivist cultural norm where fertility is highly valued. The gap between personal ambition and the reality of post-hysterectomy infertility can significantly exacerbate anxiety and stress [24].

As with many studies, using a cross-sectional design poses certain limitations. This approach captures data at a single point in time, which precludes the ability to establish causation and limits the assessment of long-term psychological consequences of hysterectomy. Additionally, the cross-sectional design does not account for potential variations in psychological outcomes across different stages of recovery, such as the acute phase immediately post-surgery versus the long-term phase (e.g., more than 1–2 years post-surgery*).* Therefore, the generalizability of the study findings should be approached with caution. Furthermore, the lack of baseline data on stress, anxiety, perception of support, body appreciation levels, and sexual problems before the study, may have influenced the interpretation of the findings. However, we believe the results of our study are robust because they are based on a large sample size. We have only included those who can read and write in Arabic; hence illiterate women’s input is not presented in this study. We have only assessed the views of women in the collectivist society of one country. It would be valuable to expand the study methodology to other collectivist countries and even to countries with a more individualistic perspective to improve understanding of women’s reactions to undergoing hysterectomy for benign conditions.

## Electronic supplementary material

Below is the link to the electronic supplementary material.

Supplementary Material 1

## Data Availability

The data that supports the findings of this study are available from the first authors upon request.
