# Supplementary material for: Prevalence and predictors of anxiety and stress among Jordanian women following hysterectomy: an observational multicentre study
Source: BMC Psychol. 2025 Mar 26;13:305. doi: 10.1186/s40359-025-02623-1 (PMC11948969; doi:10.1186/s40359-025-02623-1)
Supplement: Supplementary file 1 — Supplementary Material 1 [file 40359_2025_2623_MOESM1_ESM.docx]

**Supplementary Material**

**Table 1**: Scoring System for the Subscales of the DASS-21

**Description**: This table provides the scoring ranges and corresponding severity levels for each subscale of the DASS-21, including Depression, Anxiety, and Stress.

| Scoring system for the subscales_DASS-21 | | |
| --- | --- | --- |
| Depression subscale  0 to 9 = no symptoms,  10 to 13 = mild,  14 to 20 = moderate,  21 to 27 = severe,  28 and above = extremely severe. | Anxiety subscale  0 to 7 = no anxiety,  8 to 9 = mild,  10 to 14 = moderate,  15 to 19 = severe,  20 and above = extremely severe. | Stress subscale  0 to 14 = no stress,  15 to 18 = mild,  19 to 25 = moderate,  26 to 33 = severe,  34 and above = extremely severe. |
